# Systemic Lupus Erythematosus: Definitions, Contexts, Conflicts, Enigmas

**DOI:** 10.3389/fimmu.2018.00387

**Published:** 2018-03-01

**Authors:** Ole Petter Rekvig

**Affiliations:** ^1^Department of Medical Biology, Faculty of Health Sciences, University of Tromsø, Tromsø, Norway

**Keywords:** systemic lupus erythematosus, syndrome, anti-dsDNA antibodies, criteria, definitions, enigma

## Abstract

Systemic lupus erythematosus (SLE) is an inadequately defined syndrome. Etiology and pathogenesis remain largely unknown. SLE is on the other hand a seminal syndrome that has challenged immunologists, biologists, genetics, and clinicians to solve its nature. The syndrome is characterized by multiple, etiologically unlinked manifestations. Unexpectedly, they seem to occur in different stochastically linked clusters, although single gene defects may promote a smaller spectrum of symptoms/criteria typical for SLE. There is no known inner coherence of parameters (criteria) making up the disease. These parameters are, nevertheless, implemented in The American College of Rheumatology (ACR) and The Systemic Lupus Collaborating Clinics (SLICC) criteria to classify SLE. Still, SLE is an abstraction since the ACR or SLICC criteria allow us to define hundreds of different clinical SLE phenotypes. This is a major point of the present discussion and uses “The anti-dsDNA antibody” as an example related to the problematic search for biomarkers for SLE. The following discussion will show how problematic this is: the disease is defined through non-coherent classification criteria, its complexity is recognized and accepted, its pathogenesis is plural and poorly understood. Therapy is focused on dominant symptoms or organ manifestations, and not on the syndrome itself. From basic scientific evidences, we can add substantial amount of data that are not sufficiently considered in clinical medicine, which may change the paradigms linked to what “The Anti-DNA antibody” is—and is not—in context of the imperfectly defined syndrome SLE.

## Introduction

This study represents an open-minded approach to try to understand the nature of the syndrome Systemic lupus erythematosus (SLE), how it is defined, and how to comprehend its pathogenesis and biomarkers. The core of this approach is that it seems difficult for relevant basic and clinical scientists to agree to conformed definitions of the syndrome.

Systemic lupus erythematosus is an historically old disease described already in antiquity. The disease is a scientifically challenging (1, 2), problematic (3–6), inspiring (7, 8) and seminal (9–11), clinical syndrome (12). The syndrome is real in its existence—although hidden behind obstacles, cumbersome for patients and clinicians, and rebellious for scientists. It has inspired medical and basic biological scientists that focus on molecular biology, basic immunology, immunopathology, clinical science, genetics, and epidemiology. Scientists belonging to all these disciplines attempt to describe the nature of the syndrome SLE but also of individual parameters that constitute criteria characterizing the syndrome.

From a wider perspective, studies of anti-dsDNA antibodies in SLE have significantly enriched our knowledge about more general aspects of the immune system itself. For example, studies of SLE have promoted a better insight into how the immune system controls discrimination between anti-self and anti-non-self responses. This includes the role of the innate immune system in autoimmunity (13–16), the regulation of B cell and T cell tolerance and deletion (17–19) and receptor editing in B cells (20–24). Yet, the nature and origin of the anti-dsDNA antibody itself remain largely enigmatic. We are today not able to explain why these antibodies appear in something called SLE. On the other hand, problems to define SLE has been a concern for empirical and system sciences (25) and has been applied to nearby all aspects of the disease (7–9, 26–39).

Systemic lupus erythematosus and its biomarkers have been and are still investigated by an interdisciplinary scientific field that combine elements from empirical, basic, and clinical sciences. Historical descriptions represent an origin for empirical arguments to describe SLE as a serious disease with cutaneous manifestations (40–43). Herbernus of Tours (916 AD) was among the first to use the term “lupus” to characterize this disease [see Ref. (43)]. Further descriptions were in the nearer past expanded by the pioneering studies of Osler and Kaposi who extended our insight into the disseminated nature of lupus erythematosus; the involvement of other organs than the skin [see, e.g., Ref. (40, 44)].

**Figure 1:**
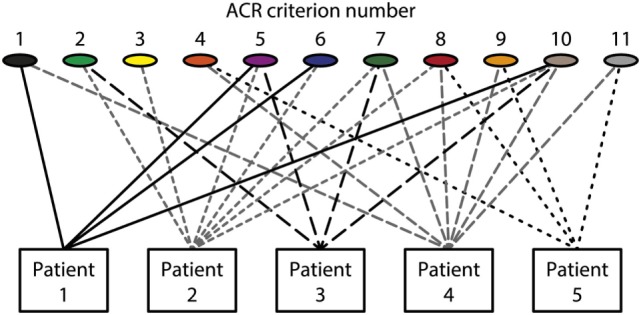
Patients classified to have systemic lupus erythematosus (SLE) by the The American College of Rheumatology (ACR) classification criteria—diversity of the clinical phenotypes. On top of the figure, each of the 11 ACR criteria is presented symbolically (see Table 1 for details on the ACR criteria). Five patients are demonstrated. The patients share some criteria, but diverge with respect to others. This chaotic figure demonstrates that the use of criteria is dubious to investigate pathogenesis of the syndrome and to search for biomarkers to characterize the syndrome SLE. How can we determine common features or biomarkers, when SLE presents so many different phenotypes? The patients in this figure are fictive but they reflect problems with the ACR in real life.

The different empirical, clinical and experimental approaches to understand what SLE is, does not imply that individual aspects are implemented in systemic multidisciplinary approaches. This statement is formulated simply because results from basic sciences relevant for SLE are only halfheartedly implemented in clinical contexts. This is discussed in detail subsequently with a focus on what SLE and “The anti-dsDNA antibody” are—and what they are not. In my opinion, there is a lack of critical cross-talks between the different fields of science that are applied to, or relevant for SLE. Many approaches deals with cohort studies based on classification criteria [see, e.g., Ref. (33, 45–47)]. Per definition, patients that fulfill a minimum of classification criteria are implemented in a cohort. This means that the patients compared to each other are phenotypically different (see Figure 1 for principle problems). This is a problematic situation.

Without implementing principles evolving from system analyses, which mean to unify interdisciplinary fields that attempt to study complex natural (48) or, e.g., medical syndromes (49), we may be left with SLE as we know it today, an enigmatic and a controversial, unclassified syndrome. Without a systematic approach, this will preclude a holistic view on the complexity of SLE as a functional or formal unit.

Unfortunately, instead of being concerned with complete systems, there is today a clear trend toward studies of SLE by individual disciplines unlinked from each other. As a paradox, many disciplines are engaged in the search to understand SLE, although the different disciplines hardly communicate with each other. Thus, we miss an organized approach to prioritize a holistic perspective by not taking all aspects of the syndrome SLE into account. This can only be achieved by concentrating on the interactions between its different elements. System science (25, 49) in our context provides a framework in which assessment of data generated by experts in different fields can be combined, and confronted with each other in order to determine what we agree on, what must be done, and what the best strategy forward must be. The following discussion is an attempt to underscore the need for profound cross-talks between scientific disciplines.

## Defining SLE: Do We Make Simplifications by Ignoring Problems That Do Not Fit into the Syndrome as an Entity?

Contemporary problematic situations have precipitated the trivial view that SLE is a multiorgan disease with poor understanding of its pathogenesis (2, 9). How can we think of SLE as a well-defined syndrome when it presents with hundreds of different phenotypes (defined by combined classification criteria)? And how can we search for biomarkers for such a poorly defined syndrome? Werner Heisenberg, who was a principal scientist in the German nuclear energy project during World War II, and a Nobel Prize laureate, formed the following anti-positivistic idiom that, I think, can be applied to all kinds of complex scientific problems. This includes also definition and understanding of syndromes like SLE: “The positivists have a simple solution: the world must be divided into that which we can say clearly and the rest, which we had better pass over in silence. But can anyone conceive of a more pointless philosophy, seeing that what we can say clearly amounts to next to nothing? If we omitted all that is unclear, we would probably be left with completely uninteresting and trivial tautologies” (50). And in this context, it is also relevant to cite Ludvik Fleck, a polish microbiologist and philosopher. He developed a system of the historical philosophy and sociology of science: “For the current state of knowledge remains vague when history is not considered, just as history remains vague without substantive knowledge of the current state” (51). Here, Fleck points to, and reminds us of an important element of system science; the implementation of empirical and historical knowledge.

The two paradigms cited earlier will function as backdrops for the problems discussed subsequently related to try to understand what SLE is. Obviously, there are needs to develop new hypotheses and to test them critically. To do so, we then have to consider one objective in sight; how do we define a hypothesis that may enable us to understand the substance of a syndrome like SLE? Here we have to ask the central questions: When are data proving something beyond subjective interpretations and simplifications, and when do we accept tautologies in order to simplify our research and paradigms related to SLE? These questions are closely associated with the term hypothesis. What is a hypothesis, and what purpose will the hypothesis serve; An approach to search for truth (the ideal context)? Or to confirm contemporary or historically simplified models (the historical or subjective context)?

## The Syndrome SLE: Historical and Contemporary Contexts

Systemic lupus erythematosus has been studied intensively since the last century (since 1942, about 65,000 SLE-related articles appear on PubMed with the search term “systemic lupus erythematosus”). This enormous amount of data and paradigms has not provided us with profound consensus on its nature, etiology or pathophysiology [see, e.g., Ref. (52, 53)]. Therefore, unclear or contradicting data and results in the past force many of us to choose solutions as if they are real although they are based on tautologies that may simplify our interpretations—leaving its significant historical context in silence (40–43). Thus, it may still be difficult to define SLE, as it was in historical, and yet in contemporary times. It seems that in antiquity, the disease was characterized by serious cutaneous affections while in modern times, more and more parameters and criteria are added to the list making up the SLE phenotypes. This makes it difficult to comprehend the nature of the disease. Here, I will discuss what we understand of SLE in terms of its wide definition as a syndrome, and if it is a possible task to use such a definition to determine biomarkers that characterize it, or point to it.

## SLE: A Multi-Organ Disease or a Disease Linking Anti-dsDNA Antibodies and Exposed Chromatin to Nephritis and Dermatitis?

Systemic lupus erythematosus is described as a multiorgan, though mysterious disease (2, 5, 6, 9, 54). The different organ and laboratory manifestations are confusing since they have no inner pathogenic coherence, but can appear in a non-concurrent way. Still it is defined as a syndrome. Then, how can we think of SLE as a well-defined syndrome when it presents with quite different phenotypes [see, e.g., Ref. (55)], and how can we search for biomarkers in such a diffuse and non-stringent situation? We do not see radical solutions in the near future as to how to explain its nature. Rather, we make simplifications, worryingly in line with what Heisenberg stated, in trying to understand the disease, and to find its biomarkers. Today we classify SLE by sets of criteria, like The American College of Rheumatology (ACR) (6) and The Systemic Lupus Collaborating Clinics (SLICC) (5) criteria, and search for biomarkers in situations where minimum requirements of criteria are fulfilled irrespective of which of the criteria are present. In this situation, the current state of knowledge remains vague, and does not take into account descriptions back in antiquity as being a serious cutaneous disease probably involving the kidneys as the malignant element. This is deduced from the fact that lupus-associated kidney and skin affections may have a common or similar pathogenic origin(s) (7, 34, 56, 57). The same problems relate to treatment; we classify a disease phenotypically as a syndrome, but we treat the most serious organ manifestations, not the syndrome as a whole [see a discussion in Ref. (58)]. Are we in fact disseminating the core of the classical disease into a myriad of parameters, biomarkers, symptoms and statistics (manuscript in progress)? Regardless, it seems that the classification systems for SLE are established, and used in diverse contexts; diagnostics, search for biomarkers, but also for single manifestations, and for therapeutics. There are obvious needs to develop new hypotheses to describe SLE!

For example, one approach would be to analyze expression levels of factors indispensable for chromatin metabolism *in vivo*. Recently, a familiar form of SLE was described. This was linked to a null mutation of the gene that encodes the secreted deoxyribonuclease DNase 1L3 (59). Sisirak et al. nicely confirmed the link between experimental DNase 1L3 deficiency in mice and a consequent autoimmunity to dsDNA and nephritis (60). Thus, the clinical version of the DNase 1L3 deficiency (59) is directly copied by the DNase 1L3 deficiency in experimental mice. This is an important approach to describe functional defects leading to pathogenic autoimmunity caused by single gene defects. More such murine models are expected to appear in the near future where deficiencies of single genes that appear central for chromatin metabolism may result in a lupus-like phenotype. If, like in the DNase 1L3 deficient mice, the clinical phenotype is characterized by anti-dsDNA antibodies and nephritis, this would give a hint to the need for re-classification of the human SLE into a “hot” SLE with an anti-dsDNA-antibody-driven chromatin-mediated nephritis phenotype. This would leave non-nephritis/non dermatitis behind as lupus phenotypes. Such studies are awaited.

## How Do We Develop Testable Hypotheses Aimed to Describe SLE?

Kuhn (61) argued that a “paradigm determines the kinds of experiments scientists perform, the types of questions they ask, and the problems they consider important” [cited in Ref. (62)]. Thus, according to Kuhn, a paradigm may form the bases for different hypotheses. Unfortunately, these may promote evolution of incommensurable models to explain the nature of the disease or the study-object. This can be anticipated as far as different hypotheses raised to solve a problem release different experimental models that may result in divergent interpretations, simply because different hypotheses are tested by different analytical approaches. These yield different analytical results and consequently, different models may appear (see subsequently for details). On the other hand, a hypothesis is formed to describe a process that may be real (as relevant for SLE), or to describe a phenomenon that lacks scientific evidence for its very existence, like the scientific history of the assumption and subsequent prove for the existence of the Higgs boson (63). In fact, the history of SLE is paralleling the history of the Higgs boson—do we lack formal evidence for the existence of the syndrome called SLE, or is SLE still formally an abstraction?

In a biological context, rather than in, e.g., theological or philosophical contexts, it is required that we can test a hypothesis by scientific methods that materialize its real biological and explainable existence. A critical hypothesis is therefore the basis to help us solve complicated system-related biological aberrations like those encountered in SLE.

One fundamental hypothesis could be formulated with the aim to study why manifestations like the classification criteria in SLE appear in various clusters, like criteria in the ACR (Table 1) and SLICC (Table 2) classification systems do. This may result in one of two possible answers; there is no causal or biological link between them; or the clusters are based on biological processes that form a causal reason for this linkage. We are far from knowing the truth about SLE, its nature, and its heterogenic phenotypes.

**Table 1:** 1997 American College of Rheumatology SLE Classification Criteria^a^ (6).

Malar rash: butterfly shaped rash across cheeks and nose Discoid (skin) rash: raised red patches Photosensitivity: skin rash as result of unusual reaction to sunlight Mouth or nose ulcers: usually painless Arthritis (non-erosive) in two or more joints, along with tenderness, swelling, or effusion. With non-erosive arthritis, the bones around joints don’t get destroyed Cardio-pulmonary involvement: inflammation of the lining around the heart (pericarditis) and/or lungs (pleuritis) Neurologic disorder: seizures and/or psychosis Renal (kidney) disorder: excessive protein in the urine, or cellular casts in the urine Hematologic (blood) disorder: hemolytic anemia, low white blood cell count, or low platelet count Immunologic disorder: antibodies to double stranded DNA, antibodies to Sm, or antibodies to cardiolipin Antinuclear antibodies (ANAs): a positive test in the absence of drugs known to induce it

*^a^Requirements: Any combination of four or more of 11 criteria, well documented at any time during a patient’s history, makes it likely that the patient has SLE (specificity and sensitivity are 95 and 75%, respectively)*.

**Table 2:** The Systemic Lupus International Collaborating Clinics (SLICC) classification criteria for Systemic lupus erythematosus^a^ (5).

CLINICAL CRITERIA
**Acute cutaneous lupus or subacute cutaneous lupus**

**Acute cutaneous lupus:** lupus malar rash (do not count if malar discoid), bullous lupus, toxic epidermal necrolysis variant of SLE, maculopapular lupus rash, photosensitive lupus rash (in the absence of dermatomyositis) **Subacute cutaneous lupus:** nonindurated psoriaform and/or annular polycyclic lesions that resolve without scarring, although occasionally with postinflammatory dyspigmentation or telangiectasias

**Chronic cutaneous lupus**

Classic discoid rash localized (above the neck) or generalized (above and below the neck), hypertrophic (verrucous) lupus, lupus panniculitis (profundus), mucosal lupus, lupus erythematosus tumidus, chillblains lupus, discoid lupus/lichen planus overlap

**Oral ulcers or nasal ulcers**

Oral: palate, buccal, tongue
Nasal ulcers
In the absence of other causes, such as vasculitis, Behcet’s disease, infection (herpesvirus), inflammatory bowel disease, reactive arthritis, and acidic foods

**Nonscarring alopecia**

Diffuse thinning or hair fragility with visible broken hairs, in the absence of other causes such as alopecia areata, drugs, iron deficiency, and androgenic alopecia

**Synovitis involving 2 or more joints**

Characterized by swelling or effusion OR tenderness in 2 or more joints and at least 30 min of morning stiffness

**Serositis**

Typical pleurisy for more than 1 day OR pleural effusions OR pleural rub Typical pericardial pain (pain with recumbency improved by sitting forward) for more than 1 day OR pericardial effusion OR pericardial rub OR pericarditis by electrocardiography In the absence of other causes, such as infection, uremia, and Dressler’s pericarditis

**Renal**

Urine protein–to-creatinine ratio (or 24-h urine protein) representing 500 mg protein/24 h OR red blood cell casts

**Neurologic**

Seizures, psychosis, mononeuritis multiplex (in the absence of other known causes such as primary vasculitis), myelitis, peripheral or cranial neuropathy (in the absence of other known causes such as primary vasculitis, infection, and diabetes mellitus), acute confusional state (in the absence of other causes, including toxic/metabolic, uremia, drugs)

**HEMOLYTIC ANEMIA**

**Leukopenia (<4,000/mm3) or lymphopenia (<1,000/mm3)**

Leukopenia at least once: In the absence of other known causes such as Felty’s syndrome, drugs, and portal hypertension Lymphopenia at least once: in the absence of other known causes such as corticosteroids, drugs, and infection

**Thrombocytopenia (<100,000/mm3)**

At least once in the absence of other known causes such as drugs, portal hypertension, and thrombotic thrombocytopenic purpura

**Immunologic criteria**

(1) ANA level above laboratory reference range (2) Anti-dsDNA antibody level above laboratory reference range (or 2-fold the reference range if tested by ELISA) (3) Anti-Sm: presence of antibody to Sm nuclear antigen (4) Antiphospholipid antibody positivity, as determined by Positive test for lupus anticoagulant False-positive test result for rapid plasma reagin Medium- or high-titer anticardiolipin antibody level (IgA, IgG, or IgM) Positive test result for anti–2-glycoprotein I (IgA, IgG, or IgM) (5) Low complement (C3, C4, or CH50) (6) Direct Coombs’ test (in the absence of hemolytic anemia)

*^a^Requirements: ≥4 criteria (at least 1 clinical and 1 laboratory criteria) or biopsyproven lupus nephritis with positive ANA or anti-DNA*.

## The Current Definition of the Syndrome SLE and Problems Linked to It

Systemic lupus erythematosus is a syndrome without a clear definition of what it is, and it is unclear whether the use of the term syndrome should at all be used in the context of SLE. The word syndrome descends from the Greek word σύνδρομον, that concisely translate into the word “concurrence” in the sense of the simultaneous occurrence of symptoms, events or parameters that are timely appearing together through a common etiology or cause (64). This strict definition of a syndrome as a condition with simultaneously appearing events is not implemented when we discuss SLE as a syndrome. In fact, the proposed ACR SLE classification is based on 11 criteria. For the purpose of identifying patients in clinical studies, a person shall be said to have SLE if any 4 or more of the 11 criteria are present, serially or simultaneously, during any interval of observations (Table 1). Thus, the manifestations do not have to appear timely together, they may appear also in an accumulated fashion one by one, and then later make up a syndrome that fulfill a term analogous to the idiom syndrome [see, e.g., Ref. (6) and discussion in Ref. (1)]. But this is not harmonizing with the classical use of the term “syndrome” (=concurrence). We are therefore facing two principally different problems: (i) the definition of the syndrome SLE and (ii) its lack of a unifying or inciting pathophysiological explanation. So we have to make a distinction between a disease with secondary manifestations, and a syndrome with (non-) concurrent manifestations that are linked in a yet not understood way—if biologically linked at all. This may be further problematized by the following perceptions using anti-dsDNA antibodies as biomarkers regarded as specific for SLE.

Systemic lupus erythematosus is per definition composed of divergent organ manifestations and laboratory aberrancies This may open for a heterogenic population of SLE patients included in cohort studies [see, e.g., Ref. (5, 9, 11, 22, 53)]. There is no common denominator for the large varieties of this disease’s phenotypes (see the principle Figure 1). In ACR criteria (Table 1), 4 out of these 11 criteria must be fulfilled to classify a disease as SLE. This means, by random combinations of 4/11 criteria, we have to accept that SLE by these criteria may theoretically present 330 different clinical phenotypes! The more recent SLICC SLE classification system (Table 2) has not helped us here. Therefore, SLE has many phenotypes that by reciprocal comparisons are quite different, is characterized by different distinct organ/laboratory manifestations, and present quite different clinical pictures. How then can we search for biomarkers correlating with the syndrome SLE as it is defined today?

## The Anti-dsDNA Antibody as Biomarker in SLE: Poor Definition of the Partners

Antibodies to dsDNA are claimed to be associated with, and to serve as biomarkers for SLE (8, 65, 66). However, “The anti-dsDNA antibody” is not an unambiguous parameter (7, 8, 52), and SLE is not an unambiguously defined disease. How then can “The anti-dsDNA antibody” serve as a biomarker for the syndrome SLE?

Theories, models or algorithms are defined as incommensurable if they derive from contrasting experimental or theoretical contexts although aimed to describe the very same problem. Their basic parameters may not be sufficient to permit scientists to directly compare the models or to cite empirical evidence favoring one theory over another (51). Incommensurable models inevitably promote scientists to be confused about terms, contexts and consequences, as is the case for SLE. With respect to SLE and to, e.g., lupus nephritis, divergent pathophysiological models may preclude consensus on pathogenesis [see, e.g., Ref. (61)]. To harmonize models that are divergent in order to reach *de facto* consensus may be a *sine qua non* in development of causal therapies.

In this context, we have to accept that facts simply are the case, subsequently interpreted as that—objective, physically distinguishable traced cases. They are discovered through proper observations from experimentally testable realities. Fleck would here submit that facts are invented or interpreted—not discovered. One can add to the problem of “conclusive facts or data” the following paradox in serious science: If experiments are performed to prove a hypothesis, then data can be interpreted as if the model simply reflects the exact fact. A problem in this context is the traditional impediment to generate experiments aimed to actively prove the opposite; namely that the hypothesis is wrong. This situation envisages how the same study-object promotes incommensurable models because those that will prove the validity of a hypothesis describes a model that differs from alternative models that are based on experiments instigated by other hypotheses.

How then can we succeed when the efforts are aimed to explain a syndrome with so many clinical phenotypes, and how can we search for biomarkers like “The anti-dsDNA antibody” in this landscape? Do we here see the contour of serious problems linked to cohort studies were the study-objects (here SLE patients) are classified by internationally well-accepted criteria (like ACR or SLICC)? In this context, it is clear that the cohort is basically heterologous and not suitable for causal and penetrating studies of SLE. Can a search for biomarkers help us here?

## SLE and Anti-dsDNA Antibodies: Do the Latter Reflect the First?

In order to discuss the nature and role of anti-dsDNA antibodies as biomarkers or pathogenic factors in clinical medicine, it may be wise to shortly describe the history of the antibody.

## Anti-dsDNA Antibodies—A Short History

In the history of immunity, hardly any naturally produced auto-antibody has attracted so many basic- and clinical-oriented scientists as the antibody against mammalian B helical DNA. During the last 40 years, more than 2,200 articles are published (PubMed search term: anti-dsDNA antibodies). The anti-DNA antibodies were first described in 1938 and 1939 in bacterial infectious contexts (67–69), and further studied and their presence confirmed 15 years later (70). In 1957, anti-DNA antibodies were described in a strict autoimmune context; in SLE (71–74).

In 2015 (7) and in 2016 (8), manuscripts were published with apparently opposite views regarding the clinical and biological impact of anti-dsDNA antibodies. In the first, their impact as biomarkers for SLE were questioned and critically discussed (7, 66). In the other, they were after a sound and critical survey of the literature defined as quintessential biomarkers for SLE (8). The clear statement denoting anti-dsDNA antibodies as strong biomarkers for SLE (8) is, however, difficult to comprehend in light of the definition of SLE, its many different phenotypes, and the wide range of biological properties and unique specificities of “The anti-DNA antibody” [a term used in the classification criteria for SLE (5, 6)]. On the other hand, the view that hesitates to accept anti-dsDNA antibodies as biomarkers for SLE (7) is hard to understand in light of the enormous efforts and data to prove exactly that. The ACR criterion 10 says: “Anti-DNA: antibody to native DNA in abnormal titer” (6), while in the SLICC criteria anti-dsDNA antibodies is described as valid if the Anti-dsDNA antibody level is above laboratory reference range (or twofold the reference range if tested by ELISA) (5).

**Figure 2:**
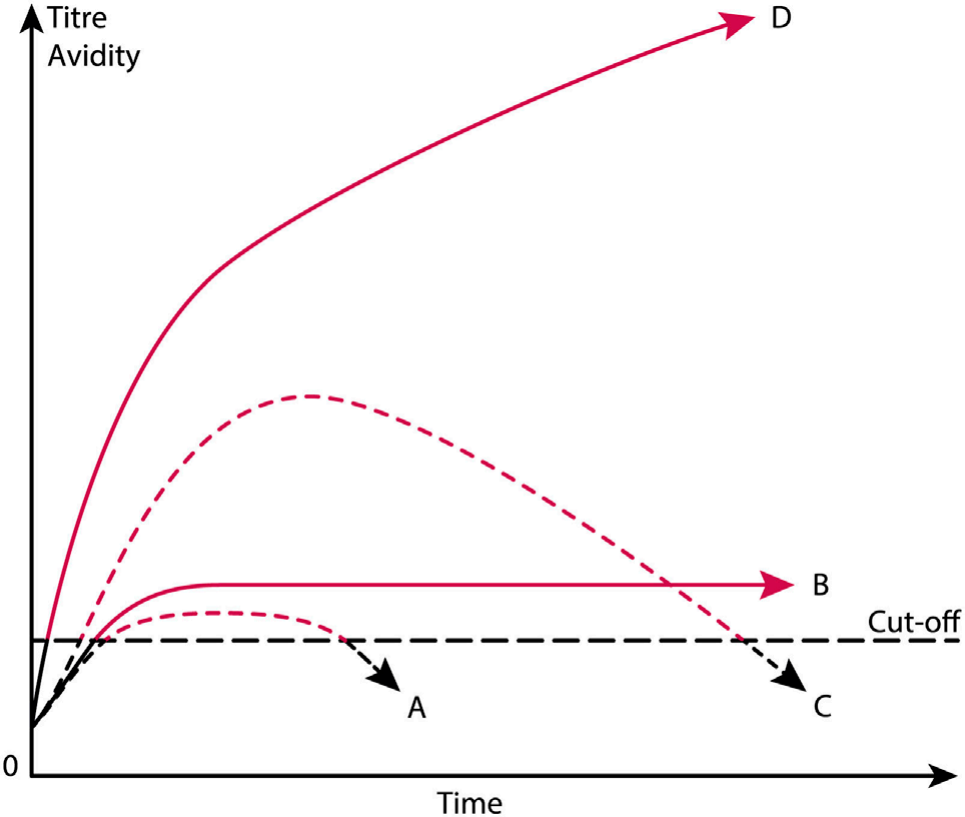
Theoretical anti-dsDNA antibody profiles in context of systemic lupus erythematosus (SLE) classification criteria. The American College of Rheumatology (ACR) and SLICC SLE classification criteria include anti-dsDNA antibodies as a criterium. As a criterium the antibodies are poorly defined. For example, a short-lived stimulus by an infectious agent may induce transient antibodies at low titers **(A)**. If the infectious stimulus prevails, the anti-dsDNA antibody may prevail at low titers, even though above the assay cutoff level **(B)**. The anti-dsDNA antibody production in **(C)** is transient, although at high titers, as a consequence of a strong, transient stimulus either of autologous or, e.g., infectious origin. In **(D)**, the immune response is characterized by sustained production of anti-dsDNA antibodies at high to very high titers. The red parts of each profile represent autoantibody levels above the antibody cut-off levels as defined by ACR or SLICC criteria. The curves are fictive and constructed empirically in order to demonstrate the variability of anti-dsDNA antibody profiles, all of which fulfill requirements in the ACR and SLICC classification criteria for SLE. See text for details.

There are several practical and theoretical tribulations linked to these definitions. The anti-dsDNA antibody is a poorly defined term that does not take into account that anti-dsDNA antibodies comprise a large array of unique specificities, quite different and unique origins, some based on spontaneous autoimmunity, some linked to cancers, others linked to external factors like drugs and infections. Furthermore, according to the classification criteria, the antibody may be weak (although above a threshold value), transient, sustained at high or low titers in different patients suffering from, e.g., SLE, cancers or infectious diseases (see the principles in Figure 2), and they may differ in avidity and cross-reactivity as long as they bind dsDNA. Still, they are accepted as a SLE classification criterion. This will be discussed in detail subsequently.

## Anti-dsDNA Antibodies—Status of Their Definition and Clinical Impact

Dogmas say that anti-dsDNA antibodies are real—they exist (although they may be induced by non-dsDNA immunogens), they occur in SLE, and they represent a classification criterion for SLE. These dogmas can, however, be analyzed in light of the philosophical view cited earlier by Heisenberg and thus be transformed and applied to our present problem. Our way to describe SLE practically represents a brave circumvention of problems or facts that do not fit into the simple solution (in the context expressed by the anti-positivists); namely that “the anti-dsDNA antibody” is not linked to SLE. Anti-dsDNA antibodies are detected in many other conditions, but the antibody may still be a pathogenic factor in SLE, provided DNA is exposed *in vivo* (7, 8, 66). The antibodies may recognize all nucleic acid structures presented in the chromatin, both in its resting state and in structures related to activation of chromatin (75, 76). These structures include DNA sequences, ssDNA, dsDNA, B dsDNA, Z dsDNA, elongated, or bent dsDNA [(77–82), reviewed in Ref. (7)]. It has never been determined if the manifold of anti-dsDNA antibodies are all pathogenic. This problem has a strong impact on choice of DNA used as targets in clinical assays if we know which of the structures are important in a clinical context.

Already after the 1938/1939 and the 1957 observations on anti-dsDNA antibodies, a growing conflict ascended when scientists tried to describe the origins of the antibodies [infectious immunity versus true autoimmunity see, e.g., Ref. (7, 27) for discussions] and their clinical impact [in diagnostic and pathogenic contexts (5, 8, 54, 66)]. In particular, the antibodies have after 1957 been surrounded by myths, conflicts and enigmas up to contemporary times, due to the problem to determine (i) their biological origin—chromatin/dsDNA or crossreactive non-dsDNA structures; (ii) if they represent one antibody population or a heterogenic mixture of DNA-reactive antibodies with different precise specificities for unique DNA structures, and (iii) if targeted and inciting DNA structures originate from different species like viruses, prokaryotes, or eukaryotes. These aspects will be discussed in detail subsequently.

In this discussion, we need to challenge the canonical impact of “The anti-dsDNA antibody” as biomarker for the autoimmune syndrome SLE. “The anti-dsDNA antibody” exists and is described in context of bacterial and viral infections (27, 67–69, 83–93), different cancer forms (94–107) and in autoimmune syndromes like autoimmune hepatitis (108), Sjøgren syndrome (109), SLE (33), or primary antiphospholipid syndrome (110) and other disorders. The term “anti-dsDNA antibody” must therefore be changed to “anti-dsDNA antibodies,” also in light of the different DNA structure-specificities that characterize the diverse anti-dsDNA antibodies. In a blinded study, it was found that assessment of anti-dsDNA antibodies by different assays was not reliable as a diagnostic tool in unselected patients with rheumatic symptoms (111, 112). Furthermore, anti-dsDNA antibodies had low positive predictive value for the SLE diagnosis [discussed in Ref. (52)]. For non-SLE patients, anti-dsDNA antibodies seemed to represent a poor predictor for SLE within an observation period of 5 years (111, 112).

Thus, as mentioned previously it can be stated that the ability to produce anti-dsDNA antibodies is not restricted to SLE. For example, normal mice respond to nucleosome-peptide immunization by producing anti-nucleosome antibodies, anti-ssDNA and anti-dsDNA antibodies, some of which may have pathogenic effects *in vivo* (4, 83, 86–88, 113–117).

## The Anti-dsDNA Antibody—Lack of Consensus Structure for DNA Used as Target Antigen in Clinical Assays

Anti-dsDNA antibody assay principles and nature of the assay targets are not recommended or specified in the ACR or SLICC criteria. Thus, we have not developed classification criteria for anti-dsDNA antibodies used in clinical analyses. We need here to define stringent structural criteria for the anti-dsDNA antibody assay targets. These must be combined with consensus on specific antibody profiles (transient versus persistent, Figure 2) and structural specificities (dsDNA, ssDNA, viral, plasmid, or elongated or bent mammalian dsDNA). Notably, the nature of target nucleic acids used in assays differ from laboratory to laboratory, which may result in detection of quite different nucleic acid structures and hence of different antibody specificities, unknown origins, or of different pathogenic impacts. For example, some of these antibodies are easy to induce experimentally, while some derive from processes yet not understood, as in SLE (7, 80, 118, 119), or exert differences in specificities for, e.g., B dsDNA versus Z dsDNA (80). It is not even settled whether autologous or heterologous instigating stimuli impose antibodies with similar or identical specificities as to those produced in SLE, although experimental data may indicate that [(87, 115, 117, 120, 121), reviewed in Ref. (7, 8)].

## Anti-dsDNA Antibodies—Origins and Clinical Contexts in a Broader Sense

Anti-DNA antibodies can, as stated earlier be induced in different contexts and clinical situations. Some of the antibodies are clinical epiphenomena (e.g., due to low avidity, or because the targets for the antibodies are hidden or not exposed *in vivo*). Others may serve as quasi biomarker for SLE, but occur in many other disorders as well (7, 52, 66, 122). Some may serve as pathogenic factors mostly in kidneys (3, 7, 57, 123–128) or in skin (56, 123, 129).

In the next section, models and evidences will be presented and discussed that may make distance to the idiom that anti-dsDNA antibodies are biomarkers for SLE. As a devil’s advocate^1^, I will turn this statement up-side-down and argue that SLE is not consistently defined, and anti-dsDNA antibodies are not confined to SLE, but to many quite different conditions. Some of these conditions will be described subsequently and serve to demonstrate that anti-dsDNA antibodies are not unique for SLE. The main statement is that the anti-dsDNA antibodies are produced transiently or permanently, and all are accepted as ACR/SLICC criteria (Figure 2). The antibodies may be specific for different species DNA (e.g., from mammalia, fungi, bacteria, and viruses), and are produced in context of diverse malignancies. They present surprisingly different specificities for shapes exposed by the whole universe of dsDNA structures as they appear in relaxed and activated chromatin (7, 75). Still they are accepted as criteria for SLE!

## Bacterial Infectious-Related Immune Responses to Nucleic Acids—The Hapten-Carrier Model

Antibodies to nuclei acids have been known for 80 years in bacterial infectious contexts, while known for 60 years in an autoimmune context. Thus, anti-DNA antibodies were described distinctively earlier than their discovery in SLE in 1957. These findings implied that DNA stimulated the immune system during bacterial infections, and in context of SLE. Still, we do not know the molecular and cellular processes that promote the production of the antibodies in SLE, while we understand more about mechanisms that impose anti-DNA antibodies in context of infections [discussed in Ref. (7, 8, 27, 130)]. The different infectious models provide central concepts to describe autoimmunity to dsDNA, and to non-dsDNA proteins contained within chromatin (for details, see subsequently).

**Figure 3:**
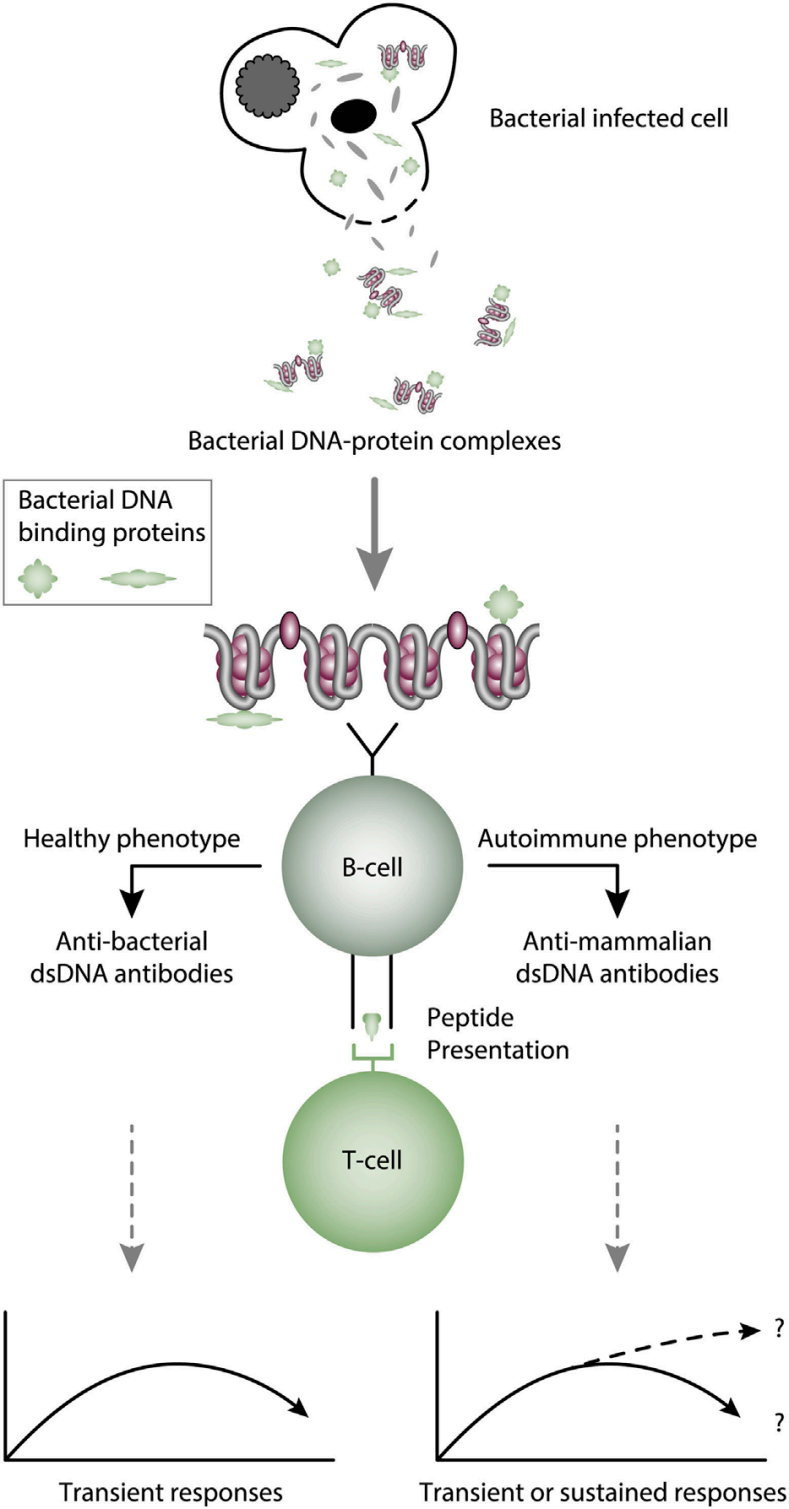
Cognate interaction of DNA-specific B cells and bacterial-derived peptide-specific T cells. This example describes a classical hapten-carrier-like model to explain production of anti-dsDNA antibodies in non-SLE (left panel) and in SLE conditions (right panel). In this model, chromatin-associated dsDNA functions as a non-immunogenic hapten that is recognized by the B cell antigen receptor, while heterologous, bacterial DNA-binding protein-derived peptides function as carrier proteins that activate peptide-specific T helper cells. In this scenario, T cell tolerance for nucleosomes is maintained intact, and the immune response is transient and is limited to the duration of the bacterial infection. According to Pisetsky et al. (83, 135), the immune response is dichotomous in the sense that in a normal immunogenic context, the antibodies recognize bacterial DNA (left part of the panel), while in an autoimmune (SLE-like) context antibodies are also produced that recognize mammalian dsDNA (right part of the panel). These processes may be operational *in vivo* in experimental and native contexts.

**Figure 4:**
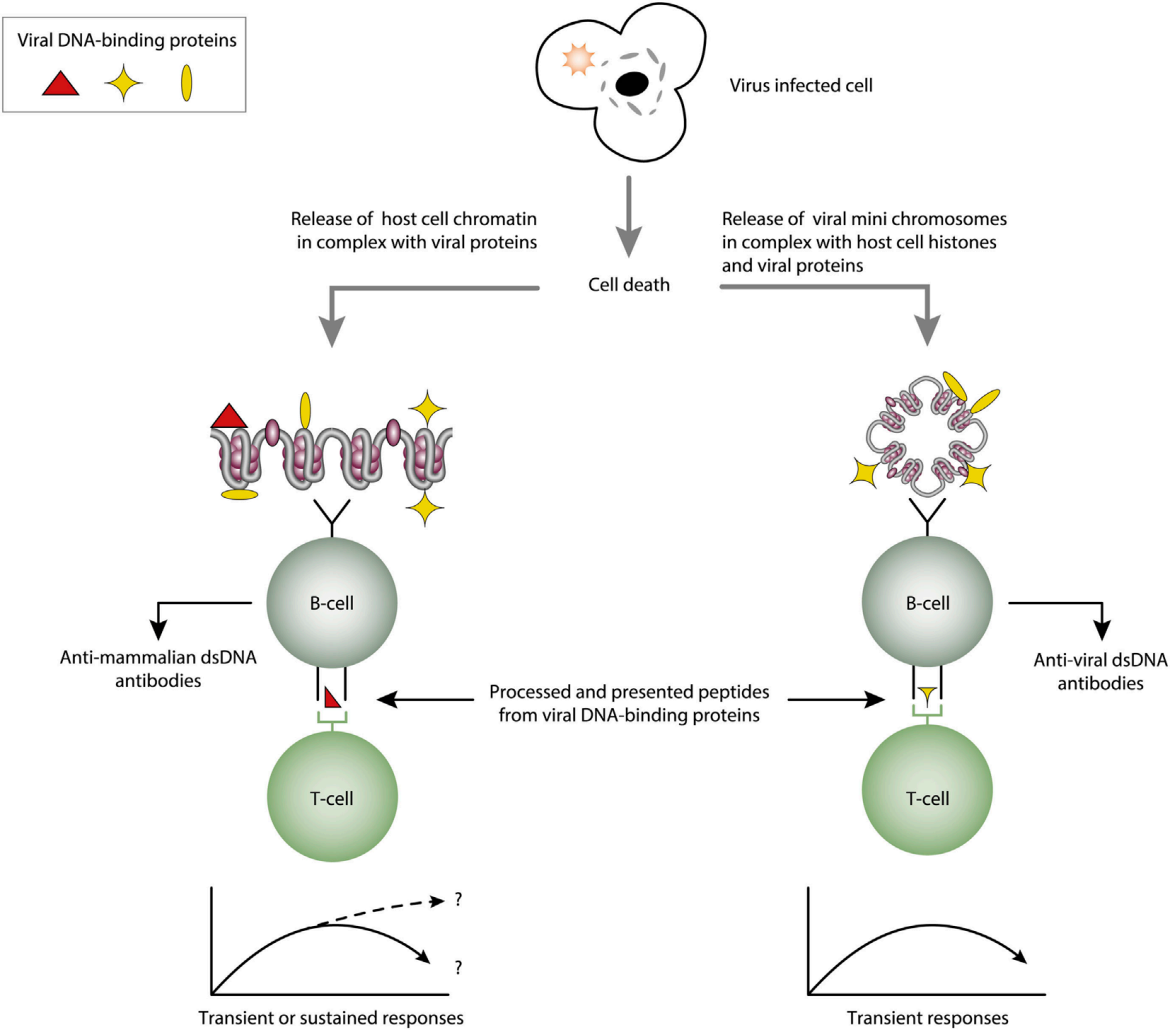
Cognate interaction of DNA-specific B cells and DNA-binding and virus-derived peptide-specific T cells. The figure presents two variants of a classical hapten-carrier-like model. In this model, chromatin from virus-infected cells are released in complex with DNA-binding viral proteins (left panel). Cognate interaction of mammalian dsDNA-specific B cells and virus peptide-specific T cells result in production of antimammalian dsDNA-specific antibodies. This process may be operational in genetically normal individuals (115, 117). In the right panel, the virus-infected cells also release viral mini chromosomes in complex with viral proteins. B cells recognize viral DNA, and present processed DNA-bound virus-encoded peptides to non-tolerant T cells. In this situation, antivirus DNA antibodies are produced. The antibody profiles depend on the time-line and kinetics of the virus infection. From this, lupus-like anti-dsDNA antibodies may appear in non-lupus individuals, thus questioning the validity of pure detection of anti-dsDNA antibodies as a SLE classification criterion.

The infectious paradigm to explain incitement of anti-dsDNA autoimmunity, changed dramatically in mid 1990s by the pioneering and important studies of Pisetsky et al. (27, 83, 85). They successfully induced immune responses to bacterial, but also to autologous mammalian dsDNA. They did so by coupling bacterial DNA to the immunogenic carrier molecule methylated bovine serum albumin (mBSA), often used to induce antibodies to different natural and synthetic DNA structures [see, e.g., Ref. (77, 78, 131–133), reviewed in Ref. (7)]. This approach was in general known as the “hapten-carrier” paradigm. It says that nucleic acids or chromatin fragments serve as a B cell-specific non-immunogenic hapten-like structure. The carrier protein was immunogenic and presented to cognate T helper cells by the nucleic acid-specific B cells [(85, 87, 88, 131, 134), see examples of hapten-carrier models in Figures 3 and 4]. This type of cognate B cell and T cell interaction resulted in humoral responses against DNA structures recognized by the B cell.

Notably, bacterial DNA, in contrast to mammalian DNA, contains immune-stimulatory structures characterized by CpG motifs or immune-stimulatory sequences [ISS, discussed in Ref. (136, 137)]. These are characterized by the presence of two 5′ purines, an un-methylated CpG motif and two 3′ pyrimidines (138). The presence of a CpG motif in bacterial DNA stimulates the secretion of proinflammatory cytokines and therefore functions similarly to an adjuvant (136, 139, 140).

Pisetsky et al. observed that immune responses to bacterial DNA–mBSA complexes presented a remarkable dichotomy pattern. While responses to bacterial DNA in normal, non-autoimmune mice were dominated by antibodies specific for bacterial DNA (Figure 3, left panel) (141), the same immunization regime in young lupus-prone mice resulted in accelerated appearance of antibodies against mammalian dsDNA—i.e., for autologous DNA typical for the enigmatic antibodies appearing in SLE (Figure 3, right panel) (83, 142). This means that bacterial infections may promote production of lupus-like anti-dsDNA antibodies on certain genetic backgrounds. Since SLE is disposed for infections (143–146) the operational mechanism to produce anti-dsDNA antibodies in SLE may therefore well be linked to the infectivity state of the patients, at least for some of them (145, 147). From this, there is no doubt that bacterial infections have the potential to promote production of anti-bacterial and anti-mammalian dsDNA antibodies [discussed in Ref. (7, 8)]. Still, the model described by Pisetsky et al. is the most adequate explanation to describe at least initiation of immune responses to dsDNA in a natural *in vivo* situation related to SLE.

Marion et al. observed and described at the same time another infectious-related mechanism that could instigate production of anti-dsDNA antibodies. They did the important observation that mammalian dsDNA in complex with the DNA-binding peptide Fus 1 derived from Tryanozoma cruzii had the capacity to induce antibodies to mammalian dsDNA (87, 148). Furthermore, they demonstrated that these induced antibodies were nephritogenic, as the immunized, normal non-autoimmune mice developed a kidney disease that was very similar to lupus nephritis. The studies described earlier represent significant seminal experiments that have helped to understand processes that may explain also spontaneous production of lupus-like anti-dsDNA antibodies. These data may also demonstrate that anti-mammalian dsDNA antibodies is not an integrated part of SLE since they can be observed in conditions other than SLE.

## Virus Infectious-Related Immune Responses to Nucleic Acids—The Hapten-Carrier Model

Viruses have been discussed as pathogenic factors in SLE for decennials (88, 149–153). This discussion refers to two possible roles of the viruses. One is that viruses may promote autoimmunity during productive infection, where the viral transcriptional factor is expressed and bind viral and host cell DNA/chromatin (115, 154–156). In dying cells, chromatin-viral transcription factor complexes are released and presented to the immune system in context of a hapten-carrier analog [see, e.g., Ref. (88, 115)]. The other role of viruses is linked to sustained productive infections, a situation that may promote sustained autoimmunity [discussed in Ref. (88, 157)]. Inspired by the Pisetsky observations, we observed a similar dichotomous response when we immunized mice with linearized polyomavirus dsDNA in complex with a carrier-protein. Normal mice produced antibodies to viral dsDNA (158), while applying the same immunization regime on young lupus-prone mice, they produced antibodies to both viral and mammalian dsDNA (159, 160); i.e., results that were in agreement with the data published by Pisetsky et al. (83, 135, 161).

## Polyomavirus T Antigen: A Natural Carrier Protein for dsDNA and Chromatin in an Autoimmune Context

Since polyomaviruses obviously had the potential to induce the production of anti-dsDNA antibodies in experimental animals, this pointed at virus-encoded proteins as potential carrier molecules rendering DNA immunogenic. Indeed, in the permissive host, polyomavirus large T antigen is required for viral transcription and replication, and binds both viral and host DNA/chromatin (117, 134), and it was shown to be expressed in SLE patients (117). Thus, virus-encoded dsDNA-binding proteins could represent a non-self DNA-bound protein that served as the T cell determinant that could provide help for mammalian dsDNA-specific B cells (Figure 4, left part) and for viral dsDNA-specific B cells (Figure 4, right part) provided they processed and presented T antigen derived peptides.

**Figure 5:**
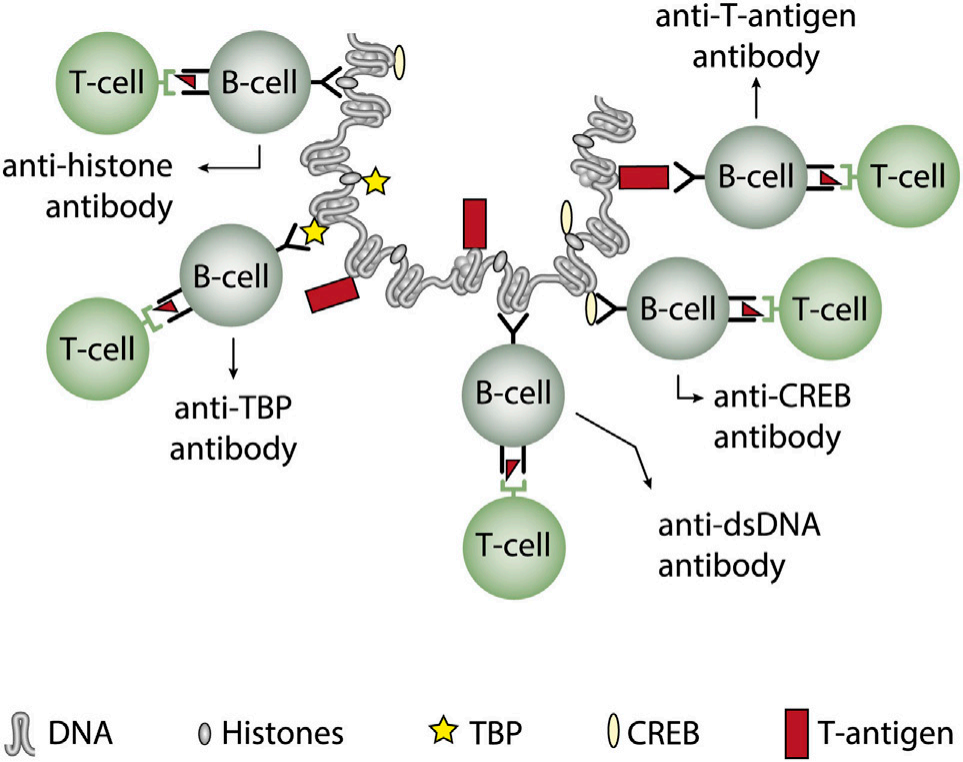
Induction of anti-dsDNA antibodies by *in vivo* expression of a single viral dsDNA-binding protein. Injection of normal mice with plasmids encoding wild type polyomavirus DNA-binding T antigen in context of eukaryotic promoters induced production of antibodies to T antigen and significant production of antibodies to mammalian dsDNA, histones, and to certain transcription factors like TATA-binding protein (TBP) and cAMP-responsive element-binding protein (CREB). All autologous chromatin-derived ligands physically linked to T antigen can therefore be rendered immunogenic to autoimmune B cells that present peptides derived from T antigen. Therefore, concerted production of autoantibodies specific for chromatin antigens, including dsDNA and histones, is not depending on a systemic lupus erythematosus background, but may appear also in quite healthy individuals.

In two experimental systems, these presumptions were verified. In one, we demonstrated that injection into normal mice with plasmids encoding wild type DNA-binding T antigen under control of eukaryotic promoters produced antibodies to T antigen. These antibodies were kinetically linked to significant production of antibodies to dsDNA, histones, and to certain transcription factors like TATA-binding protein and CREB, deduced to be produced according to the idea of the model: all autologous ligands physically linked to T antigen could theoretically be rendered immunogenic provided the presence of a (functional) repertoire of autoimmune B cells [see Figure 5 for a theoretical model based on experiments and descriptive observations (115, 117), discussed in Ref. (1)]. In this model, a diversified repertoire of chromatin-specific B cells processed and presented a single chromatin-bound viral protein. The validity of the model was further proven by the following observations. Injection of plasmids expressing irrelevant non-DNA-binding proteins like luciferase, plasmids containing T antigen sequences but lacking a promoter, or plasmids encoding and expressing a truncated T antigen without the property to bind DNA did not result in such antibodies (115). In a similar experimental system, Dong et al. (162) demonstrated that the proto-oncogene p53 can bind T antigen. When injecting *in vitro* formed complexes of p53 and T antigen, the mice responded to the immunization regime by producing autoantibodies to p53 and to T antigen, thus indicating that T antigen may render non-immunogenic autoantigens immunogenic upon complex formation. in a non-SLE condition. In other similar experiments, it has been demonstrated that immunization of normal mice with the C-terminal DNA-binding domain of the human papillomavirus E2 protein (163), and the *in vivo* expression of the Epstein–Barr virus nuclear antigen-1 (EBNA-1) in normal mice (86) both instigated the production of anti-dsDNA antibodies.

What have these experiments learned us? The infectious models are relevant to explain evolution of anti-dsDNA antibodies in SLE as well as in non-SLE conditions. Still, true spontaneous autoimmunity—i.e., driven by true autoimmune T helper cells—to dsDNA and chromatin is poorly understood, but the results discussed earlier clearly demonstrate that anti-dsDNA antibodies is a reflection of immune responses to dsDNA and chromatin in many different situations, and they can therefore principally not be a biomarker for SLE. Notably, we are today not able to distinguish between anti-dsDNA antibodies produced as true autoantibodies in SLE from anti-dsDNA antibodies produced in other contexts.

## Molecular Mimicry

**Figure 6:**
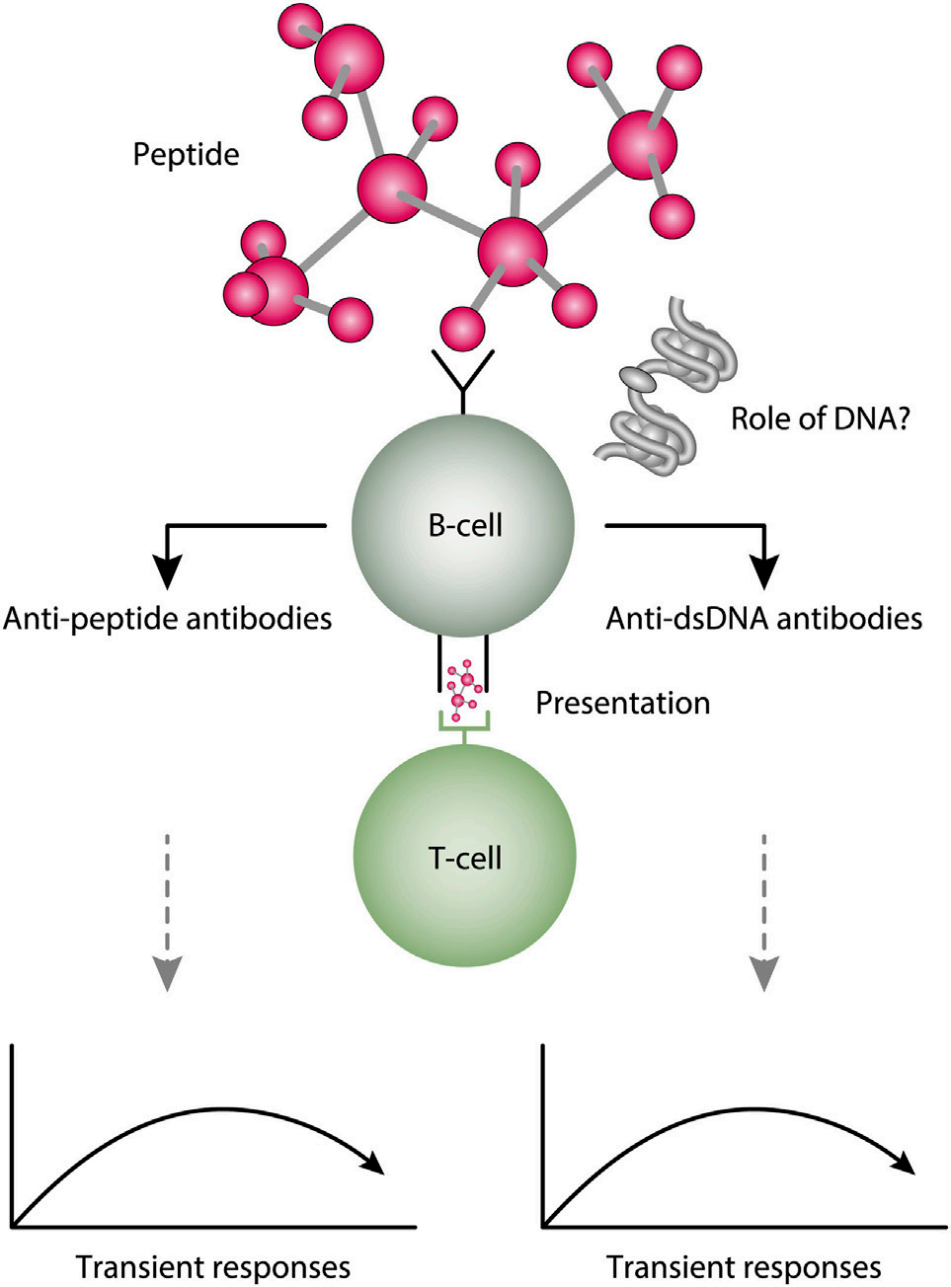
A theoretical model to explain peptide-induced anti-dsDNA and anti-peptide antibodies. Some peptides have the property to act as inducers of anti-dsDNA antibodies. There are problems with this cross-stimulating model, since it is not obvious that the peptide-induced immune response will affinity maturate toward dsDNA. Rather, somatic hypermutations in the variable heavy chain complementary determining regions (VH CDR) may shift the dual specificity toward a focused specificity for the peptide. Whether chromatin (indicated in the figure) may drive the peptide-induced anti-dsDNA antibody further is unlikely from two reasons. For the first, the initial response is controlled by peptide-specific, and not by chromatin-specific T cells. Second, if chromatin was not involved in early phases of the responses, it is no reason to believe it is rendered immunogenic in later phases of the responses.

Molecular mimicry is an alternative approach to study origin and impact of anti-dsDNA antibodies. For example, several distinct anti-dsDNA antibodies cross-react with non-nucleic acid structures like, e.g., phospholipids (164, 165), α-actinin (166–168), peptides like DWEYSVWLSN (169), entactin (113), the platelet integrin GPIIIa49-66 (170), and others. Which of the cross-reacting structures are initiating this dual immune response *in vivo* is not known. This open for the idea that the B cell recognition of dsDNA is a “by-standing” specificity which in fact has no meaning for the “real” immune response [see Figure 6, and e.g., early discussions in Ref. (93, 164), and also (171, 172)]. In this respect, it is of interest to read the manuscript of Wang et al. (173). In that review, they highlight the biological roles and structures of different reported proteins that mimic DNA. Their analytical approach might be used to discover other proteins that have this peculiar characteristic.

I do not intend in this study to discuss in depth origin and impact of anti-dsDNA antibodies that cross-react with non-DNA structures. Whether such antibodies at all should be named “anti-dsDNA antibodies” has not reached consensus, not even been principally discussed. Since this study concerns about how we define SLE, and how we define anti-dsDNA antibodies as biomarkers for SLE, it may be relevant to define three intricate difficulties related to molecular mimicry; (i) who are the initiators of the dual-specificity antibodies *in vivo*, (ii) will both specificities prevail, as the affinity maturation of their B cell heavy and light chain variable-regions prevail, and (iii) which of the structures will in the end be the *in situ* target for the antibodies. How does this translate into the understanding of avidity in context of anti-dsDNA antibodies and SLE? In a situation where an immune response is instigated by an antigen mimicking dsDNA, the primary humoral immune response will most likely produce cross-reactive antibodies. However, secondary immune responses instigated by the DNA-mimicking ligand will most probably affinity maturate toward that ligand and the antibodies may gain higher affinity toward the inducer. At the same time, the paired self-specific branch of clones may die out because they somatically mutate away for the real immunogen if not the two antigens are structurally identical. The whole story is, notably, that the immune response is instigate by the non-self antigen that engage relevant B cell clones. However, as the self antigens targeted by the crossreactive antibodies are not immunogenic, they are assumed to prevail non-immunogenic, simply because there is no reason to assume that a cross-reacting antibody may render autoantigens immunogenic. Therefore, the autoimmune branch of a cross-reaction will not influence on affinity maturation, they will turn away from the non-self branch, and they will die out.

I therefore hesitate in this study to discuss origin and impact of anti-dsDNA antibodies that cross-react with non-DNA structures. In a native situation we have to accept that we cannot say which of the cross-reacting antigens are the inducer that may prevail the immune response *in vivo*, and which one may be targeted by the antibodies. This is in the end a matter of antibody avidity and antigen availability with relevance to ask if anti-dsDNA antibodies in a deeper sense can act as a biomarker for SLE. However, the molecular mimicry model for instigating anti-dsDNA antibodies definitively show that these antibodies are not strictly linked to SLE. Most of the information from immunization experiments, and from theoretical and clinical information discussed earlier, is in disagreement with the notion that anti-dsDNA antibodies are, or can act as biomarkers for SLE. They appear in so many non-autoimmune and autoimmune situations.

## Closing Remarks

Systemic lupus erythematosus is an intriguing and engaging condition. The present human SLE paradigm evolved from being a skin disease in antiquity into a complicated syndrome involving many organs and biological processes. Although an object for many different scientific approaches, still SLE presents itself as an enigma and an abstraction difficult to comprehend in a physical and intellectual context. The studies described earlier provide insight deriving from clinical and experimental information. These have helped significantly to understand processes linked to production of anti-dsDNA antibodies in non-autoimmune and autoimmune SLE-like contexts. The data also demonstrate that anti-mammalian dsDNA antibodies are definitively not an integrated part of the syndrome SLE. They can be observed in other conditions. SLE has been described as a serious skin disease, thereof the antique name in association with a clinical morphology bringing the idea of a wolf bit, thereby “lupus erythematodes.” In modern medicine, this simple comprehensive has been left behind, and modern classification criteria have been introduced. This implies that the “lupus erythematodes” has evolved from a serious cutaneous disease, and that the seriousness implied nephritis. Nephritis was, however, not a major criterion in the antique times. In later times, lupus erythematodes has transformed from a strange, oligosymptomatic disease into a syndrome with classification criteria leaving the modern “SLE” quite different to former times disease definition. We have learned much from these paradigm shifts, about molecular biology, molecular pathology, clinical and epidemiological science, and basic and clinical immunology. In the case of our understanding of SLE, I do not think we have learnt much. It is stated that “The anti-dsDNA antibody” is a classification criterion for SLE. From all described earlier, the pure appearance of “The anti-dsDNA antibody” is closer to be an epiphenomenon in clinical medicine, rather than to be a pathogenic factor or biomarker, which, however indisputably also is associated with SLE. One potentially important question raised earlier is the following: “SLE and Anti-dsDNA antibodies: Do the latter reflect the first?” The answer to this central question is given from what is described earlier.

## Ethics Statement

The present manuscript is a review on murine and human SLE. All data are taken from original studies approved by relevant ethical committees.

## Author Contributions

The author confirms being the sole contributor of this work and approved it for publication.

## Conflict of Interest Statement

The author declares that the research was conducted in the absence of any commercial or financial relationships that could be construed as a potential conflict of interest.

## Footnotes

^1^Devil´s advocate (originally a catholic paradigm): someone who pretends, in an argument or discussion, to be against an idea or plan that a lot of people support, in order to make people discuss and consider it in more detail (Cambridge Dictionary).
